# Supplementary material for: How Acculturation Influences Attitudes about Advance Care Planning and End-of-Life Care among Chinese Living in Taiwan, Hong Kong, Singapore, and Australia
Source: Healthcare (Basel). 2021 Oct 30;9(11):1477. doi: 10.3390/healthcare9111477 (PMC8621689; doi:10.3390/healthcare9111477)
Supplement: Supplementary file 1 [file healthcare-09-01477-s001.zip › healthcare-1414147-supplementary.pdf]

Supplementary Materials

Table S1. Description of the Four Focus Group Samples

	Taiwan	Hong Kong	Singapore	Australia
Proportion of Chinese in population (%)	99	92*	76.2**	5.6
Number of focus groups	8	2	2	2
Number of participants	59	18	17	17
Gender of focus groups (female, %)	42(71.1)	10(55.5)	11(64.7)	12(70.5)

* *2016 Population By-census – Summary Results (Report). Census and Statistics Department. February 2016. p. 37.*

** *"2014 Population in Brief" Archived from the original on 13 May 2015.*

Table S2. Discussion Areas for Different Perceptions of Advance Care Planning among Chinese Living in Four Regions

	Taiwan	Hong Kong	Australia	Singapore
People with whom participants wished to discuss ACP ('who')	Participants were inclined to rely on healthcare professionals. The opinions of a doctor had to be sought before discussion with family members.	Participants wished to express their wishes to their family members and spouses during everyday life. At the end of life, prolongation of life was no longer important as the high costs of nursing care and medical treatment, limited hospital bed availability, and the wish to lighten the economic and mental burden of family members would take precedence.	Participants mainly wished to discuss ACP with their children, close family members and spouses. To avoid bringing trouble to their children, most participants had already completed their legal paperwork or were planning to do so with a lawyer or insurance company. The open attitudes of participants, which included a clear emphasis on the self and respecting each family member as an independent person, were significantly different from the attitudes of people from predominantly Chinese societies.	Governmental policies support the active promotion of will-making and implementation of ACP. Participants wished to discuss it with their relatives (spouses, children) as they had experiences with sudden deaths and did not wish to cause trouble for the younger generation.
Setting for ACP discussion ('where')	Medical institutions, outpatient clinics	The discussion could be held anywhere with no restrictions on the type of setting	The discussion could be held anywhere with no restrictions on the type of setting	The discussion could be held anywhere with no restrictions on the type of setting
Occasion for initiating ACP discussion ('when')	Occasions such as chancing upon related scenes in television programs, passing by places of religious worship, broadcasting of related news reports on audio-visual media, and experiencing certain physical symptoms may be utilised for the initiation of ACP discussion	Participant was still in good health; certain occasions are utilised for initiating ACP discussion, such as family gatherings (with avoidance of festivals and birthday celebrations) or when other family members fall sick	Participant was still in good health; the topic of ACP can be brought up naturally during everyday conversations or when participant is admitted to a long-term care facility or diagnosed with a terminal illness	The attitudes of participants were significantly more open. ACP discussion may be held at any occasion, e.g. during hospitalisation or during everyday life at home

Content of ACP discussion ('what')	*End-of-life medical treatments<br>-Participants were inclined towards receiving palliative and supportive care for the reduction of pain and increase of physical comfort<br>*Setting for end-of-life care<br>Participants preferred end-of-life care by a palliative care team at a hospital as they trusted the professionalism of hospice care personnel and did not wish to increase the burden on their children.	*End-of-life medical treatments<br>-Participants were inclined towards receiving palliative and supportive care for the reduction of pain and increase of physical comfort<br>*Setting for end-of-life care<br>Participants preferred end-of-care life at home but would opt to pass away in a hospital as the process for filing a death certificate when death occurs at home is complicated.	*End-of-life medical treatments<br>-Participants were inclined towards receiving palliative and supportive care for the reduction of pain and increase of physical comfort<br>*Setting for end-of-life care<br>Participants would be able to receive insurance pay-outs regardless of care at a hospital or at home due to long-term care benefits and robustness of healthcare schemes	*End-of-life medical treatments<br>-Participants were inclined towards receiving palliative and supportive care for the reduction of pain and increase of physical comfort<br>*Setting for end-of-life care<br>-At home: visitor restrictions at hospitals, increased convenience for relatives, related childhood experiences<br>-In a hospital/hospice facility: availability of medical equipment, ease of filing death certificate, family members will not experience fear at home
Method of ACP discussion ('how')	Similar situations faced by others could be used to initiate the subtle expression of wishes regarding end-of-life care	Participants were inclined to seek appropriate opportunities (e.g. changes in circumstances) to initiate discussion with family members during family gatherings	Direct discussion with family members to express their wishes regarding end-of-life care	Direct discussion with family members to express their wishes regarding end-of-life care
